# The prevalence of carotid plaque with different stability and its association with metabolic syndrome in China: The Asymptomatic Polyvascular Abnormalities Community study

**DOI:** 10.1097/MD.0000000000004619

**Published:** 2016-08-26

**Authors:** Anxin Wang, Lingyun Wu, Xiaoxue Liu, Zhaoping Su, Yanxia Luo, Shuohua Chen, Haibin Li, Xiangtong Liu, Lixin Tao, Jin Guo, Feng Zhang, Yibin Cao, Xingquan Zhao, Shouling Wu, Xiuhua Guo

**Affiliations:** a Department of Epidemiology and Health Statistics, School of Public Health, Capital Medical University; b Department of Neurology, Beijing Tiantan Hospital, Capital Medical University; c China National Clinical Research Center for Neurological Diseases; d Center of Stroke, Beijing Institute for Brain Disorders; e Beijing Key Laboratory of Translational Medicine for Cerebrovascular Disease, Beijing; f North China University of Science and Technology; g Department of Cardiology, Tangshan People's Hospital, North China University of Science and Technology, Tangshan; h Department of Epidemiology and Health Statistics, Academy of Public Health and Management, Weifang Medical University, Weifang; i Beijing Municipal Key Laboratory of Clinical Epidemiology, Capital Medical University, Beijing; j Department of Neurology, Tangshan Gongren Hospital; k Department of Cardiology, Kailuan Hospital, North China University of Science and Technology, Tangshan, China.

**Keywords:** carotid plaque, metabolic syndrome, stable carotid plaque, unstable carotid plaque

## Abstract

Few studies have investigated the prevalence of carotid plaque with different stability in Chinese. As is well known, carotid atherosclerosis is tightly associated with metabolic syndrome (MetS); however, the data about the association between the presence of carotid plaque with different stability and MetS was limited. The aim of our study was to investigate the prevalence of carotid plaque with different stability and its potential association with MetS in general Chinese population.

The Asymptomatic Polyvascular Abnormalities Community study is a community-based study to investigate the epidemiology of asymptomatic polyvascular abnormalities in Chinese adults. A total of 5393 participants were finally eligible and included in this study. The carotid plaque and its stability were assessed using ultrasonography. The MetS was defined using the criteria from US National Cholesterol Education Program-Adult Treatment Panel III. Data were analyzed with multivariate logistic regression models.

Of the 5393 subjects, 1397 (25.9%) participants had stable carotid plaque, 1518 (28.1%) had unstable carotid plaque in participants, and 1456 (27.0%) had a MetS. MetS was, respectively, significantly associated with the prevalence of carotid plaque (odds ratio [OR]: 1.25; 95% confidence interval [CI]: 1.07, 1.47), stable carotid plaque (OR: 1.23; 95% CI: 1.02,1.48), and unstable carotid plaque (OR: 1.27; 95% CI: 1.03,1.56) after adjusting for age, gender, level of education, income, smoking, drinking, physical activity, body mass index, low-density lipoprotein, and high-sensitivity C-reactive protein. With the number of MetS components, the prevalence of carotid plaque, stable carotid plaque, and unstable carotid plaque significantly increased (*P* for trend <0.0001), respectively.

In summary, the prevalence of carotid plaque was 54.1%, stable carotid plaque was 25.9%, and unstable carotid plaque was 28.1%. Our study revealed that the prevalence of carotid plaque, stable carotid plaque, and unstable carotid plaque was, respectively, significantly associated with MetS in the general population.

## 1 Introduction

The presence of carotid plaque, a surrogate marker of subclinical atherosclerosis and a powerful predictor of vascular outcomes, can be visualized noninvasively in the arterial wall with the use of high-resolution ultrasound, and ultrasonic measurements are often used as a surrogate endpoint in epidemiological studies on cardiovascular and cerebrovascular disease. However, few studies have investigated the prevalence of carotid plaque with different stability in Chinese.

The metabolic syndrome (MetS) currently affects more and more adult population both in developed but especially in developing countries^[1]^ and has become a major public-health challenge worldwide.^[2–6]^ It is characterized by a cluster of several cardiovascular risk factors including abdominal obesity, atherogenic dyslipidemia (elevated triglycerides [TG] and low high-density lipoproteins [HDLs]), elevations of blood pressure (BP), and raised fasting plasma glucose.^[7,8]^ Some significant studies have reported that MetS is associated with the progression of atherosclerosis^[9]^ and has become a multiplex risk factor for cardiovascular disease.^[10,11]^

Previous studies have identified that there is a correlation between MetS and the presence of carotid plaque.^[12,13]^ Northern Manhattan Study^[14]^ investigated that MetS and its components were significantly associated with carotid plaque prevalence. However, the data about the association between the presence of carotid plaque with different stability and MetS was limited in general populations in China. Therefore, the aim of our study is to investigate the presence of carotid plaque with different stability and its potential association with MetS in a Chinese community-based cohort.

## 2 Materials and methods

### 2.1 Study population

The present cohort was from the Asymptomatic Polyvascular Abnormalities Community (APAC) study, a community-based, observational, prospective, long-term follow-up study, to investigate the epidemiology of asymptomatic polyvascular abnormalities in Chinese adults.^[15]^ The detailed design and basic description of the APAC study have been published previously.^[15–17]^ Briefly, a total of 5440 participants who provided the informed consent and completed the baseline survey were finally eligible and recruited in the APAC study. The Ethics Committee of the Ethics Committees of the Kailuan General Hospital and the Beijing Tiantan Hospital approved the study, and all participants signed written informed consent. Individuals were also informed of abnormal findings and recommended treatment.

### 2.2 Data collection

From the APAC study, 5393 participants with complete information regarding MetS and carotid plaque were analyzed in this study. All participants underwent questionnaire assessment, clinical examination, laboratory tests, and carotid duplex ultrasound examinations during the baseline survey. Structured interviews with a standardized questionnaire were performed by trained investigators. The questionnaire included questions on the demographic and socioeconomic background, level of education, self-reported income, smoking, drinking, physical activity, hypertension, diabetes mellitus, hyperlipidemia, coronary heart disease, previous stroke, and about the current treatment of these diseases. Anthropometric indices included height, weight, and waist and hip circumference. Smoking was defined as at least 1 cigarette per day for more than 1 year. Drinking was defined as alcohol intake of at least 90 or 45 g of liquor per day for more than 1 year for men or women, respectively. Smoking or drinking cessation was regarded as only if it lasted for at least 1 year. The body mass index (BMI) was calculated as the ratio of body weight (kg) divided by the square of body height (m^2^). Fasting blood samples were biochemically examined for the concentration of glucose, HDL, low-density lipoproteins (LDLs), TG, total cholesterol (TC), and high-sensitive C-reactive protein (hs-CRP).

### 2.3 Definition of the metabolic syndrome

The MetS was defined using previously published criteria from US National Cholesterol Education Program-Adult Treatment Panel III. According to the definition proposed by the American Heart Association/National Heart, Lung, and Blood Institute, patients were considered to have MetS in the presence of ≥3 of the following criteria—central obesity: waist circumference >90 cm for Chinese men, >80 cm for Chinese women; a fasting triglyceride level ≥150 mg/dL (1.7 mmol/L); reduced HDL cholesterol: <40 mg/dL (1.03 mmol/L) in men, <50 mg/dL (1.29 mmol/L) in women; hypertension: systolic BP ≥ 130 mm Hg or diastolic ≥85 mm Hg or taking antihypertensive medication; and impaired fasting glucose: fasting glucose ≥100 mg/dL (5.6 mmol/L) or taking medication or previously diagnosed type 2 diabetes mellitus.

### 2.4 Assessment of carotid plaque

The complexity and advancement of carotid plaques were assessed by trained and certified sonographers using ultrasounds (Philips iU-22 ultrasound system, Philips Medical Systems, Bothell, WA). Bilateral carotid arteries were scanned with the beam focused on the near and far walls of the distal 2 cm of the common carotid artery proximal to its bifurcation. Both longitudinal and transverse images were obtained to extensively evaluate plaques. Carotid plaque was demonstrated as a thickness of 1.5 mm from the intima–lumen interface to the media–adventitia interface, or defined as a focal structure encroaching into the arterial lumen of at least 0.5 mm or 50% of the surrounding intima-media thickness value. In this study, stable carotid plaques have a uniform texture and present a smooth and regular surface, and plaques with high-level or homogeneous echoes. Whereas unstable carotid plaques were defined as plaques with incomplete fibrous cap or ulcerated plaques, and plaques with low-level or heterogeneous echoes.^[18]^ Two independent operators reviewed the carotid ultrasound examination results, and the discrepancies between their evaluations were resolved by consensus.

### 2.5 Statistical analysis

The participants were divided into 2 groups according the presence of carotid plaque. The subgroup of participants with carotid plaque presenting stable carotid and unstable carotid plaque were further analyzed. Continuous variables were described by mean ± standard deviation or median with interquartile range. Categorical variables were expressed as proportions. We used the analysis of variance (ANOVA) test for nonpaired samples of normally distributed parameters and the Kruskal–Wallis test for nonparametric variables. The χ^2^ or Fisher exact test was used for categorical variables.

The Chi-square trend test was used to test the trends of the prevalence of carotid plaque, stable carotid plaque, and unstable carotid plaque with increasing number of components of MetS. Odds ratios (ORs) and 95% confidence intervals (CIs) for the associations of MetS or the number of MetS components with carotid plaque, stable carotid plaque, and unstable carotid plaque were calculated using 3 multivariate logistic regression models. Model 1 adjusted for age and gender; model 2 adjusted for as model 1 plus level of education, income, smoking, drinking, and physical activity; and model 3 adjusted for as model 2 plus BMI, LDL, and hs-CRP. For each model, a trend test was performed after the number of MetS components was entered into the model and treated as a continuous variable. A multivariate stepwise logistic regression was also used to identify the associations between MetS and carotid plaque, stable carotid plaque, unstable carotid plaque all other variables in the model 3. Two-tailed *P* values less than 0.05 were taken to be statistically significant. All statistical analyses were carried out with SAS Version 9.4 software (SAS Institute Inc., Cary, NC).

## 3 Results

### 3.1 Patient characteristics

**Figure 1:**
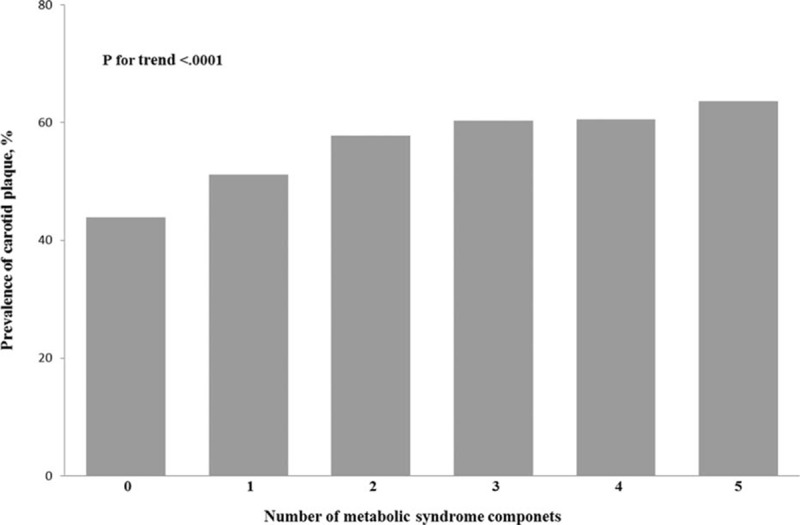
Prevalence (95% confidence interval) of carotid plaque stratified by the number of metabolic syndrome components in the Asymptomatic Polyvascular Abnormalities in Community Study (unadjusted data).

**Figure 2:**
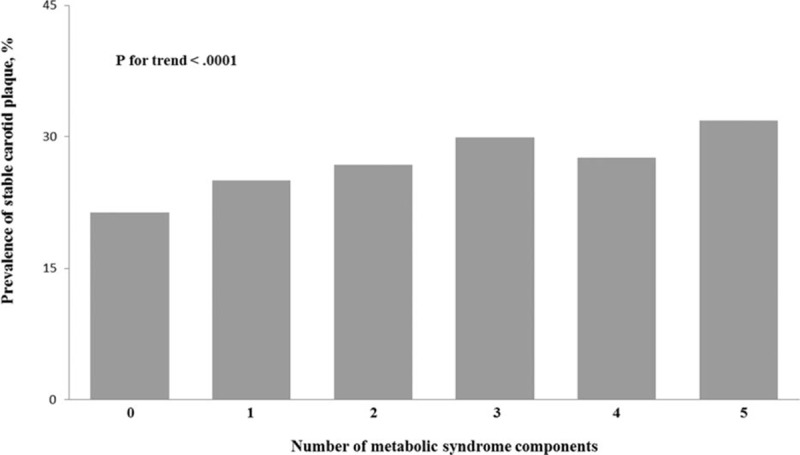
Prevalence (95% confidence interval) of unstable and stable plaque stratified by the number of metabolic syndrome components in the Asymptomatic Polyvascular Abnormalities in Community Study (unadjusted data).

**Figure 3:**
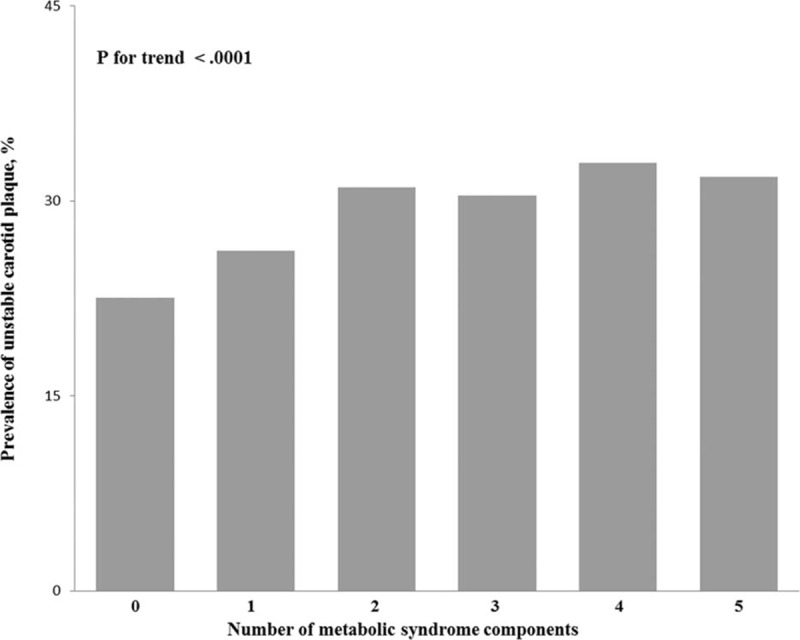
Prevalence (95% confidence interval) of unstable carotid plaque stratified by the number of metabolic syndrome components in the Asymptomatic Polyvascular Abnormalities in Community Study (unadjusted data).

Baseline characteristics of the 3 groups included in the study were presented in Table 1. Out of 5393 study participants, stable carotid plaque was detected in 1397 (25.9%) participants, unstable carotid plaque in 1518 (28.1%) individuals, and 1456 (27.0%) study participants had a MetS. There was statistically significant difference between the 3 groups in the following factors: age, gender, level of education, level of income, smoking, drinking, physical activity, blood concentration of LDL, hs-CRP, TG, TC, HDL, fasting plasma glucose, waist circumference, higher systolic BP, and diastolic BP (all *P* < 0.05). There was no significant difference between the 3 groups in BMI (*P* = 0.71). The frequency of raised TG, elevated arterial BP, raised fasting plasma glucose concentration and the degree of MetS were significantly different in the 3 groups (Table 1) (Figs. 1–3). The prevalence of carotid plaque increased significantly (*P* < 0.0001) from 43.9% (95% CI: 0.41, 0.47) in the subgroup without any MetS component to 51.2% (95% CI: 0.49, 0.54) in the subgroup with 1 component of MetS, to 57.8% (95% CI: 0.55, 0.60) in the subgroup with 2 components, and to 63.6% (95% CI: 0.51, 0.75) in the subgroup with 5 components of MetS (Fig. 1).

**Table 1:**
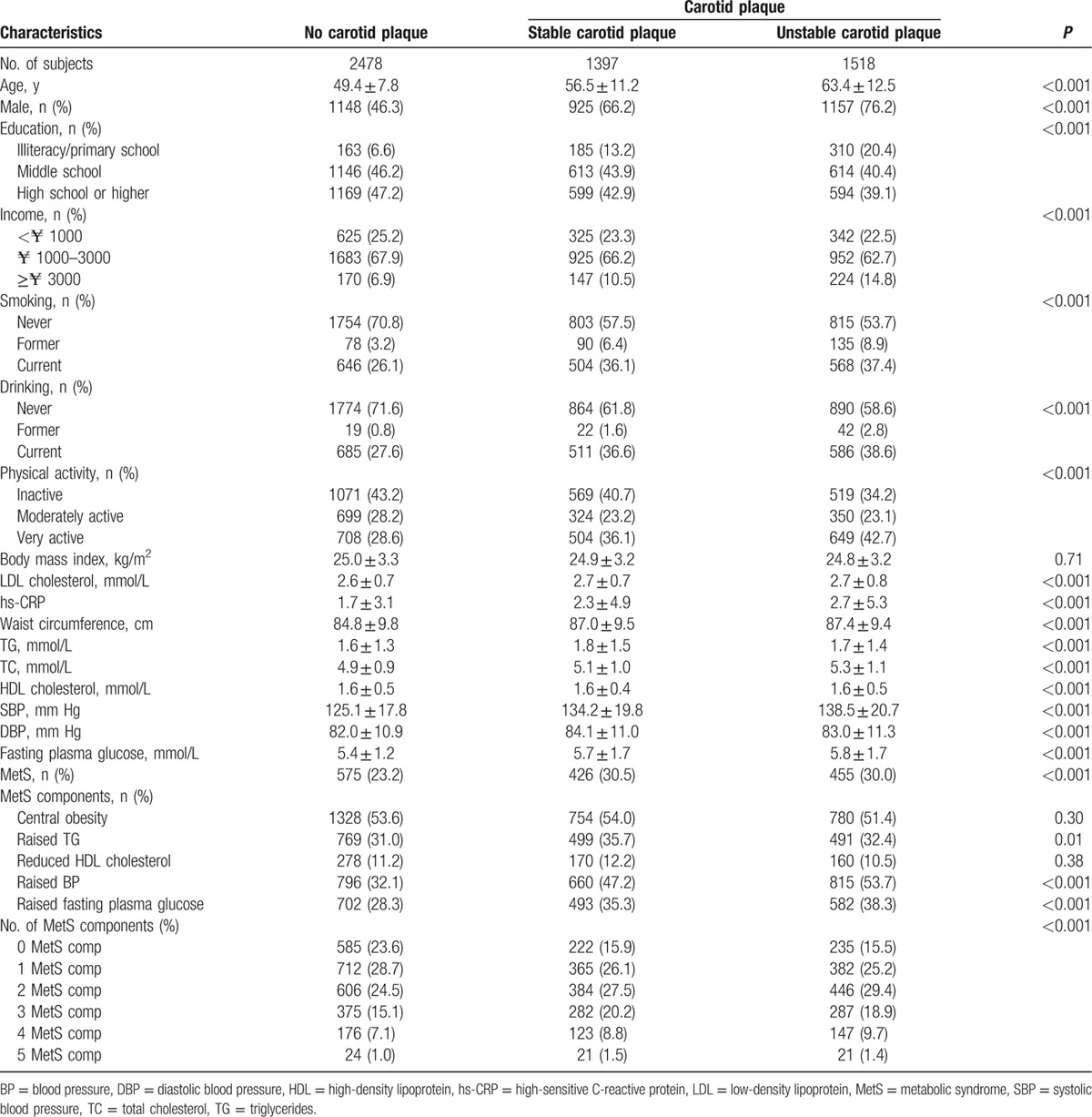
Baseline characteristics.

In the multivariate logistic regression analysis, MetS was, respectively, significantly associated with the prevalence of carotid plaque (OR: 1.25; 95% CI: 1.07, 1.47), stable carotid plaque (OR: 1.23; 95% CI: 1.02, 1.48), and unstable carotid plaque (OR: 1.27; 95% CI: 1.03, 1.56) after adjusting for age, gender, level of education, income, smoking, drinking, physical activity, BMI, blood concentration of LDL, and hs-CRP (Table 2). Using the subgroup with 0 MetS component as baseline, the ORs for the associations between the subgroups with 1, 2, 3, 4, and 5 MetS components and carotid plaque were 1.33 (95% CI: 1.09, 1.62), 1.57 (95% CI: 1.28, 1.93), 1.82 (95% CI: 1.44, 2.30), 1.96 (95% CI: 1.47, 2.62), and 2.46 (95% CI: 1.36, 4.47), respectively. The ORs for the associations between the subgroups with 1, 2, 3, 4, and 5 MetS components and stable carotid plaque were 1.38 (95% CI: 1.10, 1.73), 1.52 (95% CI: 1.20, 1.93), 1.87 (95% CI: 1.43, 2.46), 1.87 (95% CI: 1.43, 2.46), and 2.29 (95% CI: 1.16, 4.52), respectively. The ORs for the associations between the subgroups with 1, 2, 3, 4, and 5 MetS components and unstable carotid plaque were 1.30 (95% CI: 0.99, 1.69), 1.66 (95% CI: 1.27, 2.19), 1.69 (95% CI: 1.24, 2.30), 2.42 (95% CI: 1.68, 3.48), and 2.4695 (95% CI: 1.43, 6.09), respectively (model 3).

**Table 2:**
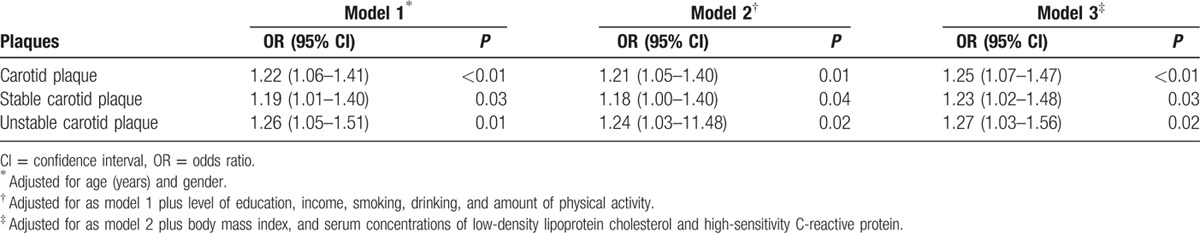
Associations between MetS presence and plaques.

In this multivariate model, the prevalence of carotid plaque including stable carotid plaque and unstable carotid plaque increased significantly (*P* for trend <0.0001) with the number of MetS components. The same held true for the 2 other models of the multivariate analysis (Table 3).

**Table 3:**
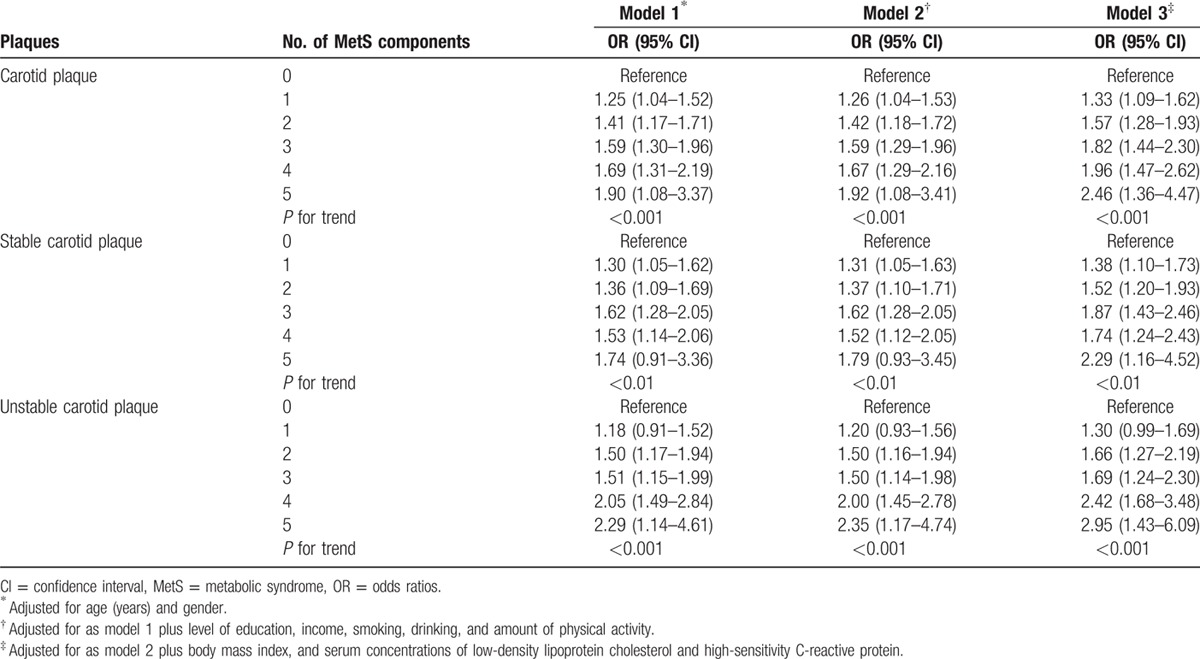
Associations between the components of MetS presence and plaques.

## 4 Discussion

In our population-based study, the prevalence of carotid plaque was 54.1%, stable carotid plaque was 25.9%, and unstable carotid plaque was 28.1%. The prevalence of carotid plaque, stable carotid plaque, and unstable carotid plaque was independently associated with both presence of the MetS and the number of components. Further, study participants with 5 MetS components had higher risk of carotid plaque, stable carotid plaque, and unstable carotid plaque than participants with 0 MetS component.

Previous hospital-based studies revealed that patients with an acute cardiovascular event were more likely to have unstable plaque in the arteries compared with patients without acute cardiovascular events.^[19–22]^ One of above studies showed that the prevalence of unstable carotid plaque in patients with an acute coronary event was 43% and those without an acute coronary event was 15%. In a European study, a certain percentage of patients with asymptomatic carotid stenosis have an unstable carotid plaque, which 78% patients had stable plaque and 22% unstable one.^[23]^ The presence of carotid plaque with different stability in general population was almost lower than community studies. As a Japanese general population study showed that the prevalence of carotid plaque was 20.0%,^[24]^ which is commonly underestimated with the reason that participants were without antihypertensive medications whose BP were less than 140/90 mm Hg. Our research results were as similar as the finding of a small sample Chinese cross-section study, which included 116 stroke-free participants and investigated that the prevalence of carotid plaque was 62.9%, stable carotid plaque was 27.6%, and unstable carotid plaque was 35.3%.^[25]^

These findings are further extended by a number of studies, which have shown both presence of MetS and its components^[13,26,27]^ to be significantly associated with carotid plaque, with increasing number of MetS components being associated with increased carotid plaque prevalence.^[28]^ The same findings have shown that individual components are the driving force behind the association between MetS and progression of plaque (>5% increase in percent atheroma volume) and not the binary presence of the syndrome itself.^[29]^ As well as few studies investigated, we further reported the association between MetS and the presence of carotid plaque with different stability in Chinese. We report that there is a strong association between MetS and its components and both stable carotid plaque and unstable carotid plaque.

As suggested in a previous study,^[30]^ hypertension and larger waist circumference were MetS components associated with atherosclerosis. One early study reported that hypertension was also identified to be an important MetS component associated with a higher prevalence of carotid plaque, as well as impaired fasting glucose.^[31]^ A majority of evidence shows that long-term elevations of BP accelerate collagen synthesis, arterial smooth muscle hypertrophy and hyperplasia, and atherosclerosis, thus increasing the development of carotid plaque.^[32]^ Abdominal obesity also have been suggested as a risk factor for accelerated atherosclerosis.^[33]^ Visceral obesity is often accompanied by impaired glucose tolerance, hyperlipidemia, and hypertension, whereas such complications are comparatively rare in subcutaneous obesity.^[34]^ Visceral fat accumulation is closely associated with the various components of MetS.^[35]^ The finding of our study was consistent with the previous study, which was reported that MetS can predict the unstable plaque.^[36]^ In addition, we further investigated that the components of Mets were strongly associated with unstable carotid plaque. As high-risk atheromatous plaque, unstable carotid plaque consists of lipid-rich atheromatous core, thin fibrous cap with macrophage and lymphocyte infiltration, decreased smooth muscle cell content, and extensive remodeling of the arterial wall.^[37]^ HDL can remove excess cholesterol from the foam cells of the evolving atherosclerotic plaque and return it to the liver.^[38]^ Patients with low concentrations of HDL cholesterol might have a high risk of plaque rupture and thrombus formation, which can lead to ischemic cerebrovascular diseases, because of the disruption of plaque.

Our study had some limitations. First, as mentioned earlier, this is a cross-sectional study so we cannot draw a causal inference which usually has to be found in a longitudinal investigation. Our findings only can conclude on an association between MetS and carotid plaque, while the causal association of MetS with carotid plaque will be tested in the follow-up study. Second, the assessment of carotid plaque with ultrasonography may be less reliable than magnetic resonance angiography or other forms of angiography. However, as an accepted method for screening carotid plaque, ultrasonography is widely used in a general population. Third, residual confounding factors could not completely be excluded, even though a stratified random sampling method was used to reduce inclusion bias. Although a stratified random sampling method by age and gender according to the data of the Chinese National Census from 2010 was used, the population in our study may not have been representative for the general population of China.

In summary, the prevalence of carotid plaque was 54.1%, stable carotid plaque was 25.9%, and unstable carotid plaque was 28.1%. The community-based study revealed that the prevalence of carotid plaque, stable carotid plaque, and unstable carotid plaque was, respectively, significantly associated with MetS.

## Acknowledgments

We thank all the participants of the APAC study for their invaluable contributions. We declare no conflicts of interest.
